# The Medical Association of Central New York

**Published:** 1907-07

**Authors:** C. A. Greenleaf


### SOCIETY PROCEEDINGS.

### The Medical Association of Central New York.

**Minutes of Thirty-ninth Annual Meeting of the Medical Association of Central New York, held in the Academy of Medicine Room in the Carnegie Library, Syracuse, N. Y., on Tuesday., October 16, 1906.**

REPORTED BY C. A. GREENLEAF, M.D., Secretary.

EDITED BY WILLIAM C. KRAUSS, M.D.

THE meeting was called to order at 10 o'clock by the president, Dr. D. M. Totman, of Syracuse, in the following words.

Members of the Medical Association of Central New York: It gives me great pleasure to welcome this association today and in this building. I would like particularly to call your attention to the library building and to offer, through the courtesy of the librarian, the unlimited use of the library during this meeting. You are all cordially invited to visit the art rooms, the library, and in fact the whole building. The secretary's report is now in order.

The secretary: Inasmuch as the secretary's report is printed in full in the Transactions, I move that the reading of it be omitted. Seconded and carried.

The treasurer's report was presented by Dr. Brown as follows:

| | | | | |
|---|---|---|---|---|
| 05 Oct. | 24 | Cash on hand | $319.39 | |
| | | Dues collected | 168.00 | |
| | | Total | $487.39 | |
| | | Disbursements | | |
| 05 Oct. | 24 | Journal Publishing Co. | | $ 28.00 |
| | | C. A. Greenleaf, stamps | | 25.00 |
| | | C. A. Greenleaf, salary | | 10.00 |
| | | C. A. Greenleaf, sundries | | 10.41 |
| | | W. C. Krauss, postage | | 3.00 |
| Oct. | 31 | F. W. Parkhurst, stenographer | | 40.20 |
| 06 June | 23 | C. A. Greenleaf, clerk hire | | 7.80 |
| July | 26 | C. A. Greenleaf, envelopes and stamps | | 25.00 |
| Sept. | 17 | A. T. Brown Printing House | | 75.00 |
| | 17 | Journal Publishing Co., | | 5.00 |
| Oct. | 15 | W. M. Brown, stamps exchange | | 4.96 |
| | | | | $234.37 |
| 06 Oct | 16 | Bal. on hand | $253.02 | |

Accepted and placed on file.

The names of the members of the committees were read by the secretary, as follows:

Entertainment.—D. H. Halstead, Syracuse; A. E. Larkin, Syracuse; Frederick Flaherty, Syracuse.

Credentials.—L. W. Rose, Rochester; C. E. Congdon, Buffalo; W S. Cheeseman, Auburn.

Business.—C. O. Boswell, Rochester; H. A. MacGruer, Syracuse; Charles E. Low, Pulaski.

Ethics.—A. A. Young, Newark; F. S. Crego, Buffalo; Elias Lester, Seneca Falls.

Publication.—Wm. C. Krauss, Buffalo; C. A. Greenleaf, Canoga; W. M. Brown, Rochester.

"President's Address" was read by Dr. David M. Totman, Syracuse. (See p. 321. January number.)

Dr. Hersey G. Locke, Syracuse, read a paper entitled "Responsibility in Mental Diseases."

Dr. Charles E. Low, Pulaski: I feel it would only be justice on the part of the business committee to introduce a resolution favoring the discussion of both these papers. I am sorry we did not have further time to devote to Dr. Locke's original and valuable paper as well as to the president's address. I therefore move that Dr. Angell be tendered the privilege of discussing these papers at this time.

Dr. E. B. Angell, Rochester: I rise to a question of personal privilege, in the first place, for myself, and in the second place for the president. In the first place, it is not usual to discuss the president's paper. I believe it should be referred to a committee, which should report this afternoon. Unfortunately I did not arrive in time to hear the president's paper, and I should be very glad to read it before discussing it. Therefore, I move that the president's paper be referred to a committee of three, to report after luncheon. Seconded and carried.

The president appointed Dr. E. B. Angell, Dr. Frederick Sefton and Dr. H. G. Locke as such committee.

Dr. Rose then read a paper entitled "Abscess of the Brain, Operation,—Death. Report of Case."

Dr. W. B. Johnson, Batavia: I would just like to say a few words about brain abscess and make some remarks about another case that I operated on. When I first saw the man he was in a condition of complete coma, and had not spoken a word or swallowed a mouthful for three days. The only symptom of focal irritation was referable to the Rolandic area. Spasmodic contractions of the levator ani were present. The man was in such a condition that I began to operate without anesthetic. I made a five-eighths inch trephine of the bone over the lower opening of the Rolandic fissure. The abscess was the size of a walnut. Then the man began to recover consciousness and we gave chloroform to finish the operation. He opened his eyes and I said "How do you feel?" He said "Bully." That was the first word he had spoken in three days. He died that night. We performed an autopsy. On taking off the skull cap I found that the abscess was at the very lowermost portion of the Rolandic fissure, and the cavity was caused as much by separating as by a destruction of the brain tissue. In the second case the peculiar feature was, that when I trephined, nine days after the injury, I removed a piece of his hat and a piece of bone. He was having all this time an epileptic form of convulsions. The lesion was on the right side of the brain. The convulsions still kept up. We started the operation without chloroform, he was so comatose. The convulsions kept up at three minute intervals. So sure was one of my associates that the lesion was on the opposite side of the brain, that I simply ran my knife across the scalp and examined the skull over the corresponding area on the other side. There was no visible lesion of the bone there, and the man got well. His epileptic symptoms ceased after three days, and he is now well, three years after the operation.

The president: We have with us today a very welcome visitor in the person of Professor Burt G. Wilder, of Cornell University. We are all familiar with the fact that Professor Wilder has been taking various brains as he can get them. I am sure I voice the sentiment of all when I extend to him the hearty welcome of this association.

Dr. Wilder then read a paper entitled "The Medical Profession and the Simplified Spelling."

Dr. William S. Cheeseman, Auburn, presented a specimen for the examination of the members of the association, and spoke as follows:

"Presentation of Specimen of Sarcoma of Kidney, weight 4½lbs., removed from a child aged twenty months." This is a tumor weighing four and a half pounds which I removed from a child twenty months old. The tumor does not represent its original size; it has shrunken in the preserving fluids until it is not more than two-thirds its original size, if it is as much. The really formidable appearance of the mass is I think shown by the sketch which I will pass around. The history of this case, to be very brief, is this: The mother first observed in this infant a varicocele of the right side. That was the first symptom. Gradually the parents became aware that the abdomen was slowly enlarging. They thought nothing of it, thinking it was an accumulation of fat, but it was not very long—in fact, the tumor at the time it was removed was only of two months' growth—until the abdomen was filled from side to side by a very large mass.

On examination this mass was elastic—I could not say fluctuating, but elastic—and of course flat from one side of the abdomen to the other, intestines being displaced to the left. The vague history led me to believe that the tumor must have swung from the right side. There was no pain. The child had fits of crying, but they seemed to be more from irritability than anything else. At no time was there found any blood or albumin in the urine. Particles of growths are often found in the urine, and that is usually a most characteristic feature. Nothing of that sort occurred in this case. The appearance of the urine was throughout entirely normal. I was strongly inclined from the very first to regard the case as one of sarcoma of the kidney. The pathology of these growths is not, I believe, very clearly laid out; that is to say, it is considerably unsettled as to the exact pathology of many of them. Some of them are mixtures of sarcoma and carcinoma, and others with other forms of growth. The greatest interest in the case to me centered in the technique of the removal of the growth. The child was thin, but not emaciated, and quite pale, but not remarkably anemic. The lips were still red.

The very first question which arose, of course, was that of the choice of access to the growth. Dr. Abbe, in his reports of several cases of extirpation of tumor, has preferred to use the side incision opening from the line of the umbilicus, around to the back. He published a photograph of the child after the recovery showing the scars. In thinking the matter over it seemed to me that incision had many disadvantages, among which was cutting of muscle fibers and weakening of the abdominal muscles. The other incision is around the front of the abdomen, and reaching the tumor from the front. I chose that incision, going through the right rectus muscle. The abdomen was split from the ribs to the pelvis through the right rectus muscle. This gave me access to the growth. Of course the tumor was there covered by the posterior layer of the peritoneum. The large intestine overlay the growth from above down. The incision was made on the outside of the large intestine in order to avoid destroying the vascular supply of the colon.

The operation in itself was not one of great difficulty, but the greatest difficulty encountered was found in the hemorrhage which occurred in separating the posterior layer of the peritoneum. It took very little pressure to control this bleeding, but as rapidly as possible the peritoneal surface was dissected off with the fingers and finally the kidney itself reached and the mass delivered through this incision. It reminded me of nothing so much as delivering the head of a fetus. It came out finally by pressure of both sides. It was a matter of no difficulty at all to remove the growth. By this time of course the child was very much shocked. I would say that the recovery was an uneventful one, to my surprise, as I really expected nothing but death. The child became ravenously hungry in a few days, and looks well now. Of course the ultimate result of this case is, I am afraid, a dark prognosis. The statistics are very discouraging as to the ultimate recovery.

Dr. William D. Johnson, Batavia, read a paper entitled "A Case of Pancreatic Cyst."

Dr. W. L. Wallace, Syracuse, read a paper entitled "Intestinal Resection; a Rapid and Safe Method."

### Afternoon Session.

The president announced that the first business of the afternoon is the election of officers for the ensuing year. It was moved and seconded that the association proceed to the election of a president.

Dr. Jacobson: It has been the custom of this society since time immemorial to elevate to the presidency the first vice-president. Dr. W. B. Jones, of Rochester, is first vice-president at the present time, and it affords me great pleasure to present his name for the office of president for the next year. Seconded by Dr. Charles A. VanDerBeek.

It was moved and seconded that the secretary cast the ballot of the association for Dr. W. B. Jones for president for the ensuing year. The secretary cast the ballot as directed and the president declared Dr. Jones elected president for the ensuing year.

The president then called for nominations for the office of first vice-president.

Dr. Krauss: I would like to nominate for first vice-president a gentleman with whom most of you are acquainted, and a man who has attended nearly all the meetings of this association, a man very popular in Buffalo and all through western New York. I refer to Dr. Carlton C. Frederick of Buffalo. Nomination seconded.

It was moved and seconded that the secretary case the ballot of the association for Dr. Frederick for first vice-president. Carried. The secretary then cast the ballot as directed and the president declared Dr. Frederick elected first vice-president for the ensuing year.

The president called for nominations for second vice-president.

Dr. Henry L. Elsner, Syracuse: I nominate Dr. Pascal M. Dowd of Oswego. Nomination seconded.

The secretary cast the ballot under instructions by vote and the president declared Dr. Dowd elected second vice-president for the ensuing year.

The president called for numinations for secretary. It was moved and seconded that the president case the ballot of the association for Dr. Greenleaf for secretary for the ensuing year. Carried.

The president cast the ballot of the association for Dr. Greenleaf for secretary for the ensuing three years.

The president called for nominations for treasurer.

Dr. C. O. Boswell: I nominate Dr. William M. Brown for treasurer for the next ensuing three years. Seconded.

Dr. Low moved that the secretary cast the ballot of the association for Dr. Brown for treasurer for three years. Seconded. Carried.

The secretary cast the ballot as directed, and the president declared Dr. Brown elected treasurer for the next ensuing three years.

The secretary read the list of names presented for membership in the association as follows:

The president: You have heard the names proposed for membership, as read by the secretary. What is your pleasure?

Dr. Henry L. Elsner, Syracuse: I move that the secretary cast the ballot of the association in favor of these candidates. Motion seconded and carried.

The president declared the candidates elected members of the association.

Dr. Thomas Jameson, Rochester, then read a paper entitled "Pyloric Obstruction."

Dr. Jones, Rochester: It is quite true that almost all hopeless cases go through a long period of suffering, of gradually aggravated symptoms, of gradually aggravated treatment, by many physicians as a rule. Nearly all the cases that do come to something more radical show by their pathology that there has been long periods during which there was a chance for relief, and in almost all of the cases, including cancer, there have been long periods in which absolute cure was possible; and the attitude of those who are responsible for this in each individual case is terribly near to that of an ignorant man who wastes precious moments. The patient comes to a table, the operation being a success in every respect technically, and yet the patient's vitality is too far exhausted for good results, living a couple of weeks, and perhaps dying of marasmus. During this period of ineffectual treatment all sorts of laboratory tests are made, if the attendance has been at all intelligent, and many times with great benefit, and also many times being entirely useless. I would like to narrate to you a case where some of the best diagnosticians in the world could not make out whether a certain case was ulcer of the stomach or possibly cancer. On the operating table it proved to be cancer. After this the diagnosis was "probably ulcer," but as a precautionary measure microscopical diagnosis was made, when it proved unquestionably cancer, and was treated as such.

Dr. Allen A. Jones, Buffalo: I am glad Doctor Jones has brought the question of the case up, because it emphasises an important phase of that which has heretofore been considered an entirely medical subject, that is, hypochlorhydria. Until within recent times medical men were quite justified in treating hypochlorhydria as a medical affection, but experience has led some of us to regard with suspicion many cases of hypochlorhydria. These cases of hypochlorhydria, some of them are extremely resistant to treatment, obstinate, not yielding, and usually lead a patient rather an irksome existence. Recently I saw one in a young man who had been suffering sometime, about a year, from the so-called hypochlorhydria, and at operation pregastric lesions were found from the pregastritis, which, upon being loosened and undergoing proper treatment completely relieved his symptoms. We did not find any thickening of the gastric wall in or near the pyloris, and therefore the stomach was not opened. The man was relieved. I have seen a number of cases with gallstones, and, on the other hand, some cases of partial stagnation—at all events, delay in the passage of the food from the stomach into the duodenum, and many gastric symptoms.

Dr. Henry L. Elsner, Syracuse: It appears to me that no subject could be presented to a body of general practitioners of greater importance than the one touched upon in this paper. If our modern methods of diagnosis establish anything in connection with gastric diagnosis it is the fact that these cases are surgical, that they should be so regarded, that the diagnosis can be made with ease, and that medical treatment avails nothing. Now, it matters little whether you have an intragastric or an extragastric stricture, there is no medicine known to the profession today which will give you a sufficent motor strength to empty that stomach and overcome the obstruction. The stomach tube, or even a thorough examination, will readily make the diagnosis; and there is no disease in which the stomach tube will aid so much as in this very condition,—obstruction at the pyloric end of the stomach. The leading symptom, the condition which is always present, is stagnation, and when stagnation persists, from whatever cause, and medical treatment, tried only a very short time, avails nothing, the case should be turned over to the surgeon.

Now, that is safe teaching. I have reached that conclusion as the result of some experience, painful at times, and I believe that the physician today must teach that as a fact just as positive as anything in medicine. It may be that the pendulum has swung too far in the direction of the surgeon, but the truth of the matter is that these benign strictures can only be treated by surgical means, and as such I believe the profession must recognise them as surgical cases from beginning to end.

Dr. I. Levy, Syracuse: I think we ought to emphasise the importance of the motor function. For a long time the stomach tube was used simply to determine the amount of hydrochloric acid. As to the question of pepsin, I believe that is of less importance than the motor question. The tube should be employed, not only in the morning when the stomach is empty, but should be used also six hours after the meal, and if stagnation exists then it is a sure sign the stomach is not emptying itself properly and there is some disturbance of the motor function.

Dr. A. B. Miller: One point Dr. Elsner failed to make clear is that while the surgical procedure is not always successful in the benign instances, the majority of the cases where they are recognised early would never reach the malignant stage. They would be relieved by the surgical procedure before malignancy intervened. In connection with the capacity of the stomach, the author of the paper took the wind out of my sails when he said his patient's stomach held a little more than a gallon. I operated on a case for pyloric obstruction where the stomach was washed out and the capacity was a pint less than a gallon. The patient said to me that had he thought if it made any difference he could have taken another pint. This man was simply a shadow, and today he is a man weighing 150 pounds. At the time of his operation there were berry seeds in his stomach. As he had not eaten any berries in ten days before that time, it showed that the seeds had been there that length of time. The shock does not seem to be severe in these cases. I reported a case before the Syracuse Academy of Medicine of an individual who simply was obliged to be carried into the hospital on a cot, unable to walk, who underwent the shock of operation. She took on flesh and was enabled to do her daily work, and even conceived after that, a year or two before her death. In that instance the stomach symptoms were entirely relieved, and instead of dying a death of starvation she died from another cause.

Dr. E. B. Angell, Rochester: The committee appointed to consider the recommendations of the president's address in relation to expert medical testimony respectfully submit: That it unqualifiedly approves of the effort to solve this difficult problem in medico-legal ethics, but that careful consideration of the method proposed compels the reluctant conclusion that it would be unconstitutional, and therefore impossible of application. The constitutional right each contestant has to present all his evidence in his own way would preclude the application of such measures as proposed in the president's address.

Your committee would suggest a thorough discussion of this important subject, to the end that the profession at large may become better acquainted with the abuses associated with the present method of introducing medical testimony, and the need for reform in the direction of a "square deal."

Respectfully submitted,
EDWARD B. ANGELL,
FREDERICK SEFTON,
HERSEY G. LOCKE.

Dr. Nathan Jacobson then read a paper entitled "Extensive Gangrene resulting from Contact with a Live Wire," and presented a case for the inspection of the society.

Dr. Henry L. Elsner read a paper entitled "Leaflets from Clinical Records."

Dr. Charles E. Low read a paper entitled "Physician—Civilian."

Dr. William C. Krauss, read a paper entitled "The Need of a Trained Microscopist in Every Community.

Dr. Charles O. Boswell read a paper entitled "The Leukocytes in Surgical Infections."

Dr. Allen A. Jones, Buffalo: The chief point, as illustrated by Dr. Boswell, is the slight value of leukocytosis in certain cases. We must not be guided by the blood findings so far as to be misled in cases. Doctor Boswell illustrated in his paper how leukocytosis was present with a gangrenous appendicitis, and it falls to the lot of most of us to see such cases. One case comes to my mind of gangrenous appendicitis with leukocytosis of nine thousand. So that a leukocytosis, while it is valuable, taken in connection with all other clinical manifestations, should not be allowed to mislead us.

Dr. M. M. Lucid read a paper entitled "Epiphyseal Separation of the Ends of the Humerus."

Dr. L. W. Rose, Rochester: The splint mentioned is not Dr. Moore's splint for the traction or extension treatment of the upper fracture of the humerus. It was designed by Dr. Swinburne, of Albany, some years ago, and is known as the Swinburne splint. From the description that Doctor Lucid gave I think that was the Swinburne splint instead of Dr. Moore's splint. He designed no splint for the upper end of the humerus.

He designed a mode of reduction for fractures of the upper end of the humerus, which was supreme abduction, and they were dressed in this position (indicating). In the matter of diagnosis and treatment of fractures of the lower end of the humerus, I think one is pretty safe in their treatment if they do not make any diagnosis, if they use an anesthetic and make supreme traction, flexion, supination and pronation. When you can do that with your elbow grasped with the other hand, and you can do it under an anesthetic, and then putting the arm in that position, without any splint at all; you do not need any splint, using the Jones method of suspension—and you will get most beautiful results. The family will be shocked and they will probably tell the neighbors that the doctor didn't think there was any fracture because he didn't put a splint on; but after taking that method of reduction and using the halter, called the Jones halter, you have traction there, and your body is a good splint. You do not need any sling—if you use a sling you will have trouble. Then you can get an *x*-ray, and no man is safe in a fracture without an *x*-ray picture. That is the only thing to protect the surgeon.

Dr. Albert L. Beahan, Canandaigua, read a paper entitled "A plea for the Earlier Diagnosis of Appendicitis."

The president: The following paper is upon the same subject, and we will listen to that before discussion is had of this paper.

Dr. Luzerne Coville read a paper entitled "A Case of Appendicitis and an Autopsy."

Dr. Jacobson: It seems to me these papers have brought up matters of interest and present a number of points we ought to emphasise at this time. In the first place, as to matter of diagnosis. Every surgeon is, so to speak, in the hands of the physician who calls him into the case, as the early diagnosis made by the medical attendant is many times responsible for the successful work of the surgeon, and a late diagnosis is quite as often responsible for the fatal termination of the case; but with all that we may sometimes be called to a case early and may not succeed in our work and have a happy termination of the case. Now, we have a certain picture before us of classic symptoms upon which we will sometimes seek to depend in making a diagnosis; but I want to say right here that we find the greatest difference between the clinical manifestations many times and the pathological conditions.

I had occasion to present this subject to our society a few years ago, and I have been impressed with that. A physician sent a case into our hospital in the spring in which practically there were no well defined acute manifestations. The tenderness was mild, the rigidity was minute, the pain was not great, the man was not vomiting. He had a history of recurring attacks of appendicitis, but we found a very high leukocyte count with a large percentage of cells. We operated and found gangrenous appendicitis. You will find many times that discrepancy, an advanced conditions of things, with a moderate clinical picture. I had occasion to operate on a child within a few weeks, who was well at midnight, taken sick about one o'clock in the morning, brought in about six, and I operated at seven, and found a strip of omentum wound around the appendix, causing the most intense manifestations. In that particular case I found a few hours made a greater difference than in an average case several days would have made. I have found that a condition that does not yield at once to medicinal treatment is a surgical case of appendicitis.

For a long number of years I was ready to admit that there was a group of cases of appendicitis that were medical, but I am growing to the conviction that I cannot tell what cases belong to that class, and that for that reason I believe that safety lies in regarding all as being surgical. Now, I cannot say with Dr. Beahan that every case of appendicitis that is unruptured and in which there is no peritoneal complications is sure to get well after operation. We see, unfortunately, this is not the case, and we cannot always determine why this is not the case, but Dr. Dowd knows of a case where we operated on a physician not long ago where we had a gangrenous appendicitis, and where everything was deemed pure, and everything progressed apparently satisfactorily for forty-eight hours, but the patient died. The autopsy did not reveal any explanation of that physician's death. The physician had been refused for life insurance a year before because of a renal condition, but there was nothing after his operation which suggested that he had any renal disease.

Dr. F. W. Sears: My experience bears out the idea that you should not let your pregnancy interfere with your operation. Your mother and child will do better before rupture. I have operated on five pregnant women, and they have stood the operation better than the non-pregnant women. I operated on one a week ago today, and she has not shown the slightest trouble. Don't allow a pregnant woman to go on to rupture; attend to it at once.

Dr. C. C. Frederick: I do not believe there is a diagnostician living when he takes a case of appendicitis that can give the course of that case for the next twenty-four hours, or forty-eight or seventy-two hours, he doesn't know anything about it. Put fifty cases right along side by side, apparently equally severe, and probably not more than two or three of the fifty will follow the same course. Therefore I say, after an abundant experience, that in the early part of an attack of appendicitis, as soon as the diagnosis is made, it is far safer to operate than it is to wait, for you never know how the case is going to go. It may turn out to be simply a catarrhal attack, you may have a rupture in forty-eight hours with a general peritoneal attack, and you never know. Get them out and then you have them out for all time, because the patient is probably going to have a recurring attack, and you have got to take it out ultimately.

Dr. W. B. Johnson: The plea for an early diagnosis is the most important plea we can make in appendicitis, and I rely most upon symptoms and an understanding of what those symptoms mean. The first symptom is pain, and the cause is the contraction of the appendix trying to rid itself of foreign material. If it succeeds, good; if not, bad. The second is nausea. That is the reflex nausea of abdominal pain. The third is tenderness, and that is the tenderness of the sore appendix, the disease in which has penetrated to the peritoneal fold. The fourth is the temperature caused by absorption. When you get these four symptoms you can operate in ninety-nine cases out of a hundred and be right. I don't dare to operate until I get temperature. You usually get it in the first twelve hours. Dr. Sears spoke of operating on pregnant women. I operated on four in one year. Not one of them aborted or had bad symptoms. Dr. Beahan speaks of having a series of cases with one death. I had sixty-four cases with two deaths, but in the next two I had two deaths, which made it four deaths in sixty-six.

Dr. William M. Brown, Rochester, read a paper entitled "Version or High Forceps?"

Dr. Snow: I don't think Doctor Brown need to apologise for the paper. It is a most excellent paper, and I desire to emphasise what he says. There is only one point where I disagree with the technique the doctor gives, and that is in the eclampsia. Formerly I lost quite a number of cases, and I realised that I lost them through inability to get the head quickly. In the cases where we get them in seven or eight months the head is large in proportion to the body. For the last three or four years I have adopted a method of dilating the cervix as well as I can and applying the forceps and delivering in that way; and I am able to say that all the children, where they have been a period of eight months or longer, you can deliver more slowly and with more safety to the child in that way, and with just as much safety to the mother, if it is done in the method the doctor has described. In only one case of eclampsia have

I used the forceps. That is a case during the present year where the woman was badly exsanguinated. After carefully thinking it out I thought I could put on my forceps quickly and manipulate the uterus less by using the forceps. As the child was dead I thought the mother would perhaps stand the forceps, and we fortunately saved the mother.

Dr. Low: Dr. Congdon wished his paper read by title. It is entitled: "Abdominal Section for Trauma of the Uterus. Puncture of non-peuperal Uterus with Prolapse of Bowel; Violent Seisure and tearing away of sixteen inches of Gut; immediate abdominal section, repair of uterus, lacerations, intestinal anastamosis, recovery."

Dr. M. E. Gregg, Elbridge, read paper entitled "Conservatism in Modern Therapeutics."

Dr. I. H. Levy, Syracuse, read a paper entitled "Achylia Gastrica."

Dr. Jones, Buffalo: The association of achylia gastrica and carcinoma is interesting. As a rule it has not been my custom to confuse achylia gastrica with carcinoma. Regarding the nervous cause of achylia, some years ago my associate, Dr. Stockton, and I called attention to the fact that many of these cases present a peculiarly aggravated atrophy. We argued that a long functional disturbance might finally lead to a structural defect. In 1893 I published a paper on this question, shortly following Einhorn's paper, and noted in that paper a few cases that were studied for some years afterwards; and before the meeting of the American Medical Association of 1897, I read a paper on the association of achylia gastrica with chronic diarrhea. The association of chronic diarrhea and achylia is interesting, and the diarrhea demands in the way of treatment the proper diet and proper medicines, taken, I mean, in relation to the gastric condition. I believe, though, that there is an associated chronic atrophic enteritis in these cases from prolonged and overworked conditions of the intestines. It is a very interesting paper.

The president: Gentlemen, I wish to thank you all for the courtesy you have shown during the day, and I bespeak for the next meeting, which is to be held in Rochester a year from now, probably a better meeting even than we had today. The meeting last year in Buffalo was one of the most successful I have ever attended of the association, and we have sought to do as well as we can by you today here in Syracuse, and I hope our efforts have not entirely failed. There being no further business to be transacted, I declare this meeting of the association closed.

Meeting adjourned to meet in Rochester next year.

